# Sublingual microcirculatory changes during high-volume hemofiltration in hyperdynamic septic shock patients

**DOI:** 10.1186/cc9271

**Published:** 2010-09-27

**Authors:** Carolina Ruiz, Glenn Hernandez, Cristian Godoy, Patricio Downey, Max Andresen, Alejandro Bruhn

**Affiliations:** 1 Departamento de Medicina Intensiva, Pontificia Universidad Católica de Chile, Marcoleta 367, Santiago 114-D, Chile; 2 Departamento de Nefrología, Pontificia Universidad Católica de Chile, Marcoleta 367, Santiago 114-D, Chile

## Abstract

**Introduction:**

Previous studies have suggested that high volume hemofiltration (HVHF) may contribute to revert hypotension in severe hyperdynamic septic shock patients. However, arterial pressure stabilization occurs due to an increase in systemic vascular resistance, which could eventually compromise microcirculatory blood flow and perfusion. The goal of this study was to determine if HVHF deteriorates sublingual microcirculation in severe hyperdynamic septic shock patients.

**Methods:**

This was a prospective, non-randomized study at a 16-bed, medical-surgical intensive care unit of a university hospital. We included 12 severe hyperdynamic septic shock patients (norepinephrine requirements > 0.3 μg/kg/min and cardiac index > 3.0 L/min/m2) who underwent a 12-hour HVHF as a rescue therapy according to a predefined algorithm. Sublingual microcirculation (Microscan for NTSC, Microvision Medical), systemic hemodynamics and perfusion parameters were assessed at baseline, at 12 hours of HVHF, and 6 hours after stopping HVHF.

**Results:**

Microcirculatory flow index increased after 12 hours of HVHF and this increase persisted 6 hours after stopping HVHF. A similar trend was observed for the proportion of perfused microvessels. The increase in microcirculatory blood flow was inversely correlated with baseline levels. There was no significant change in microvascular density or heterogeneity during or after HVHF. Mean arterial pressure and systemic vascular resistance increased while lactate levels decreased after the 12-hour HVHF.

**Conclusions:**

The use of HVHF as a rescue therapy in patients with severe hyperdynamic septic shock does not deteriorate sublingual microcirculatory blood flow despite the increase in systemic vascular resistance.

## Introduction

High-volume hemofiltration (HVHF) is a potential rescue therapy in patients with severe septic shock, and some clinical studies suggest that HVHF can decrease vasopressor requirements and improve lactate clearance [1,2]. Therefore, HVHF may have a place in refractory septic shock by contributing to the stability of systemic hemodynamics and eventually improving systemic perfusion. However, studies supporting HVHF are rather small and non-randomized, and this prevents investigators from drawing a more definitive conclusion about its real impact on clinically relevant outcomes. Indeed, decreases in vasopressor requirements and lactate levels may not necessarily reflect a real improvement in perfusion. In the past, therapies such as steroids and nitric oxide synthase inhibitors have been shown to increase vascular tone without any significant benefit in terms of perfusion or survival [3,4]. In addition, it is now well accepted that hyperlactatemia may be explained by mechanisms not related to hypoperfusion [5]. Clearly, it would be desirable to assess the impact of HVHF on perfusion determinants (particularly, on microcirculation) more directly.

The development of optical techniques such as orthogonal polarized spectral imaging and, more recently, side dark field videomicroscopy (SDF) has made it possible to visualize microcircirculation at the bedside. Microcirculation is known to be markedly compromised during septic shock and these disturbances are considered to play a central role in multiple organ failure. By means of these novel techniques, the impact of conventional therapies on microcirculation is starting to be unraveled [6-9].

There is very limited information concerning the potential effects of HVHF on microcirculation during septic shock. Only one previous experimental study has addressed this subject [10], but unfortunately, the model induced only non-severe microcirculatory derangements, making the results difficult to interpret. Beneficial effects of HVHF have been related to non-specific removal of mediators, which could potentially contribute to the reversion of microcirculatory disturbances induced by sepsis. However, the most evident clinical effect of HVHF is an increase in arterial pressure, and this occurs as a result of an increased systemic vascular resistance, and not of an increase in cardiac output, at least in hyperdynamic patients [2]. Therefore, it is critical to determine whether this increase in vascular resistance is associated with a detrimental effect on microcirculatory flow. We performed a prospective observational pilot study to assess changes in sublingual microcirculation during HVHF in patients with severe hyperdynamic septic shock.

## Materials and methods

Our local ethics committee approved the study, and informed consent was obtained from the patients or their relatives. All septic shock patients in our institution are managed with a norepinephrine-based, perfusion-oriented management algorithm. Septic patients presenting a circulatory dysfunction at the emergency department or the pre-intensive care unit (pre-ICU) are subjected to vigorous fluid resuscitation followed by central venous catheter insertion and basal measurements of lactate (Radiometer ABL 735; Radiometer, Brønshøj, Denmark) and central venous oxygen saturation (ScvO_2_). Patients who develop persistent hypotension or hyperlactatemia are transferred promptly to the ICU. The algorithm involves early aggressive source control and fluid loading followed by norepinephrine, which is adjusted to keep a mean arterial pressure (MAP) of at least 65 mm Hg. Fluid resuscitation is guided by pulse pressure variation (if the patient is already on mechanical ventilation) or by central venous pressure. Pulse pressure variation (ΔPP) is calculated as ΔPP = 100 × (PP_max _- PP_min_)/[(PP_max _- PP_min_)/2]. If after fluid optimization norepinephrine is greater than 0.3 μg/kg per min, patients are characterized as having severe septic shock. At this stage, all patients must have a pulmonary artery catheter in place and be sedated and connected to mechanical ventilation. Mechanical ventilation and sedation are managed in accordance with current protective strategies [11]. Dobutamine is indicated as an inotrope for patients with low cardiac index (CI) (less than 2.5 L/min per m^2^) or low ScvO_2 _or mixed venous oxygen saturation (SmvO_2_) values (less than 60%) not responsive to other measures and with an MAP of greater than 65 mm Hg. HVHF is indicated for patients who fail to respond to all preceding management steps, including source control and fluid optimization guided by ΔPP [2,12].

Specific inclusion criteria for this study were septic shock according to the 1992 ACCP-SCCM (American College of Chest Physicians/Society of Critical Care Medicine) consensus [13], norepinephrine requirements of at least 0.3 μg/kg per min to maintain an MAP of greater than 65 mm Hg for at least 1 hour before deciding HVHF, progressive hyperlactatemia (greater than 2.4 mmol/L and an increase in lactate levels during 4 hours of full resuscitation), and a CI of at least 3 L/min per m^2^. Patients without full commitment for resuscitation or with active bleeding or an undrained source of surgical sepsis were excluded.

All patients had a pulmonary artery catheter in place and were mechanically ventilated following current guidelines [11], with fentanyl/midazolam sedation targeted to a Sedation-Agitation Scale (SAS) score of less than 3. No patient received steroids, vasopressin, or drotrecogin alpha either before or during the hemofiltration procedure. Blood transfusions were indicated before the procedure if the hemoglobin value was less than 8 g/dL.

### High-volume hemofiltration technique

A 13.5-french double-lumen hemodialysis catheter was inserted in the femoral vein under local anesthesia (Q-plus; Covidien, Mansfield, MA, USA). HVHF was performed with a polysulfone hemofilter that had an area of 1.5 m^2^, a wall thickness of 40 μm, and an internal diameter of 200 μm (Diacap acute-M; B. Braun, Melsungen, Germany). The hemofiltration monitor was adjusted for a blood flow of 200 mL/min. During the first 60 minutes, the ultrafiltration rate was increased gradually to 100 mL/kg per hour according to hemodynamic tolerance while always keeping a neutral fluid balance (Diapac; B. Braun). Pre-hemofilter ultrafiltrate reposition was performed using a bicarbonate-based solution with the following final composition: sodium 140.0 mmol/L, potassium 2.0 mmol/L, calcium 1.5 mmol/L, magnesium 0.5 mmol/L, chloride 111 mmol/L, bicarbonate 35 mmol/L, and dextrose 1 g/L and an osmolality of 296 mOsm/L (S-BIC 35 and SH-EL 02; B. Braun Avitum AG, Glandorf, Germany). The extracorporeal system was not anticoagulated, and patient core temperature was kept over 35°C by the heating device coupled to the monitor and by warming the solutions when necessary. According to our ICU protocol [2], all patients were scheduled to receive a 12-hour period of HVHF with a single hemofilter, during which additional fluids and the norepinephrine dose were adjusted to maintain an MAP of at least 65 mm Hg and a ΔPP of less than 10%. Before the start of the procedure, all patients should have a ΔPP of less than 10%.

### Measurements

Patients were assessed before starting HVHF (baseline), after 12 hours of HVHF, and 6 hours after stopping HVHF. Each assessment consisted of hemodynamic measurements (MAP, heart rate, CI, pulmonary artery occlusion pressure, and central venous pressure), vasoactive requirements, perfusion parameters (arterial lactate, SmvO_2_, and urine output), Sequential Organ Failure Assessment (SOFA) score, and sublingual microcirculation imaging.

### Sublingual microcirculation imaging

Sublingual microcirculation was assessed with SDF with a 5× lens (MicroScan(r) for NTSC [National Television System Committee]; MicroVision Medical, Amsterdam, The Netherlands). At each time point, at least five 10- to 20-second images were recorded. After saliva and oral secretions were gently removed, the probe was applied over the mucosa at the base of the tongue. Special care was taken to avoid exerting excessive pressure on the mucosa, and this was verified by checking ongoing flow in the larger microvessels (greater than 50 μm). Analog images were digitalized by using the pass-through function of a digital video camera recorder (Sony DCR-HC96 for NTSC; Sony Corporation, Tokyo, Japan) and were recorded instantaneously in AVI format on a personal computer with the aid of commercial software (DVGate Plus 2.3; Sony Corporation).

Images were analyzed blindly and randomly using a semiquantitative method. According to recommendations of a consensus committee [14], the image analysis consisted of determinations of (a) flow: proportion of perfused vessels (PPV) and microvascular flow index (MFI); (b) density: total vascular density (TVD) and perfused vascular density (PVD); and (c) heterogeneity: MFI heterogeneity (Het MFI). Briefly, to determine MFI, the image was divided in four quadrants and the predominant type of flow was assessed in each quadrant and characterized as absent = 0, intermittent = 1, sluggish = 2, or normal = 3; the values of the four quadrants were averaged. MFI heterogeneity was calculated as Het MFI = (MFI_max _- MFI_min_) × 100/MFI_mean_. For TVD and PVD, a gridline consisting of three horizontal and three vertical equidistant lines was superimposed on the image. All of the vessels crossing the lines were counted and classified as perfused vessels (continuous flow) or non-perfused vessels (absent or intermittent flow, the latter of which is the absence of flow for at least 50% of the time). Densities were calculated as the total number of vessels (TVD), or the number of perfused vessels (PVD), divided by the total length of the gridline in millimeters. PPV was calculated as PVD × 100/TVD (percentage). Large and small (less than 20 μm) vessels were analyzed separately. According to recommendations from experts [14], the analysis of large vessels is of limited interest, and in this study they were used as a quality control to ensure that no excessive pressure was being applied on the sublingual mucosa. Therefore, all of the data from sublingual microcirculation presented correspond to small vessels.

### Statistical analysis

Data with normal distribution are presented as mean ± standard deviation, and data not normally distributed are presented as median and 25th-75th percentiles. Repeated measures analysis of variance with the Bonferroni *post hoc *test was used to evaluate changes along time for normally distributed data, and the Friedman test with Dunn test correction was used for variables without normal distribution. Correlations were determined by the Pearson coefficient or Spearman's rho for data with normal and non-normal distributions, respectively. Analysis was performed with GraphPad Prism version 5.00 for Windows (GraphPad Software, La Jolla, CA, USA). A two-sided *P *value of less than 0.05 was considered statistically significant.

## Results

Twelve consecutive patients with severe hyperdynamic septic shock (seven men and five women, 57.9 ± 13.2 years old) were recruited between March 2007 and March 2009. Baseline characteristics are presented in Table 1. The more common sources were abdominal in five and pulmonary in two. All patients started HVHF less than 6 hours after meeting the inclusion criteria. One patient had a baseline norepinephrine requirement of 0.28 μg/kg per minute, but he had met the norepinephrine inclusion criteria during the screening period (specifically, a norepinephrine dose of greater than 0.3 μg/kg per minute for more than 1 hour with a ΔPP of less than 10%). Baseline assessment was performed just before the start of HVHF. Only two patients were receiving dobutamine for at least 2 hours before the start of HVHF, and its dose was not changed during the procedure (patients 1 and 6). All patients survived until the end of the study period, but five patients died at day 28 (42%). No technical problems with the procedure were observed and no change of hemofilter was required in any patient.

**Table 1:** Baseline characteristics of patients at the start of high-volume hemofiltration

Patient	Diagnosis	APACHE II score	SOFA score	Survival (day 28)	MAP, mm Hg	NE dose, μg/kg per min	CI, L/min per m^2^	SmvO_2_, percentage	Lactate, mmol/L
1	Cholangitis	34	13	Yes	70	0.30	3	49	6
2	Necrotizing fasceitis	24	10	Yes	67	0.56	5.5	79	4.7
3	Cholangitis	25	11	Yes	65	0.50	5.3	76	8.3
4	Catheter related sepsis	31	15	No	70	0.60	3.1	71	4.1
5	Diverticulitis	19	14	Yes	75	0.37	5.5	80	2.6
6	Peritonitis	19	11	No	64	0.30	4.4	61	6.7
7	Pneumonia	21	13	Yes	74	0.50	3.1	58	2.6
8	Necrotizing fasceitis	25	13	No	64	1.00	4.8	79	4.5
9	Pyonephrosis	23	13	Yes	66	0.28	3.5	71	3.6
10	Mesenteric ischemia	23	13	Yes	62	0.62	3.4	78	2.6
11	Empyema	27	14	No	63	0.30	4.8	96	13
12	Endocarditis	25	15	No	70	0.60	3	70	5.8

Mean		24.7	12.8		67.5	0.49	4.1	72	5.4
SD		4.4	1.7		4.3	0.21	1.0	11	3.0

APACHE II, Acute Physiology and Chronic Health Evaluation II; CI, cardiac index; MAP, mean arterial pressure; NE, norepinephrine; SD, standard deviation; SmvO_2_, mixed venous oxygen saturation; SOFA, Sequential Organ Failure Assessment.

### Hemodynamic and perfusion parameters

MAP and systemic vascular resistance index (SVRI) increased and lactate levels decreased at 12 hours of HVHF, with no changes thereafter. CI, SmvO_2_, O_2 _transport, and O_2 _consumption did not change during or after HVHF (Table 2).

**Table 2:** Evolution of microcirculatory scores and hemodynamic and perfusion parameters during the study

Parameter	Baseline	After 12 hours of HVHF	6 hours after HVHF
MAP, mm Hg	67.5 ± 4.3	74.5 ± 6.8^a^	76.0 ± 9.4^a^
Norepinephrine, μg/kg per min	0.49 ± 0.21	0.44 ± 0.45	0.26 ± 0.38
CI, L/min per m^2^	4.06 ± 1.11	3.68 ± 1.36	3.55 ± 1.12
SmvO_2_, percentage	72.4 ± 1.7	71.4 ± 7.0	76.1 ± 6.0
Lactate, mmol/L	5.38 ± 2.99	3.66 ± 2.39^a^	3.64 ± 3.89^a^
IDO_2_, mL/min per m^2^	543 ± 211	483 ± 350	475 ± 173
IVO_2_, mL/min per m^2^	137 ± 63	135 ± 101	108 ± 40
O_2_ER, percentage	26 ± 12.3	27.8 ± 0.7	23.1 ± 6.0
SVRI, dyne-s/cm^5 ^per m^2^	1,027 ± 268	1,373 ± 408^b^	1,432 ± 375^b^
Hemoglobin, g/dL	10.1 ± 1.4	10.2 ± 2	10.2 ± 1.3
Core temperature, °C	38.1 ± 1	37.2 ± 0.9	37.5 ± 1.1
SOFA score	12.8 ± 1.7	13.1 ± 2.1	12.4 ± 2.5
TVD, n/mm	13.1 ± 1.9	13.6 ± 3.3	14.2 ± 3.8
PVD, n/mm	9.6 ± 2.5	11.1 ± 3.0	12.1 ± 4.3
PPV, percentage	73.6 ± 15.6	81.7 ± 13.3^c^	83.2 ± 14.7^c^
MFI^d^	2.15 (1.64-2.28)	2.5 (1.96-2.7)^b^	2.5 (2.31-2.63)^b^
Het MFI^d^	0.44 (0.36-0.47)	0.4 (0.12-0.65)	0.29 (0.18-0.32)

^a^*P *< 0.05 versus baseline; ^b^*P *< 0.01 versus baseline; ^c^*P *< 0.06 versus baseline; ^d^data are presented as mean ± standard deviation or as median and 25th-75th percentiles. CI, cardiac index; Het MFI, heterogeneity of microvascular flow index; HVHF, high-volume hemofiltration; IDO_2_, oxygen delivery index; IVO_2_, oxygen consumption index; MAP, mean arterial pressure; MFI, microvascular flow index; n/mm, number of vessels per millimeter; O_2_ER, oxygen extraction ratio; PPV, proportion of perfused vessels; PVD, perfused vascular density; SmvO_2_, mixed venous oxygen saturation; SOFA, Sequential Organ Failure Assessment; SVRI, systemic vascular resistance index; TVD, total vascular density.

### Microcirculatory parameters

**Figure 1:**
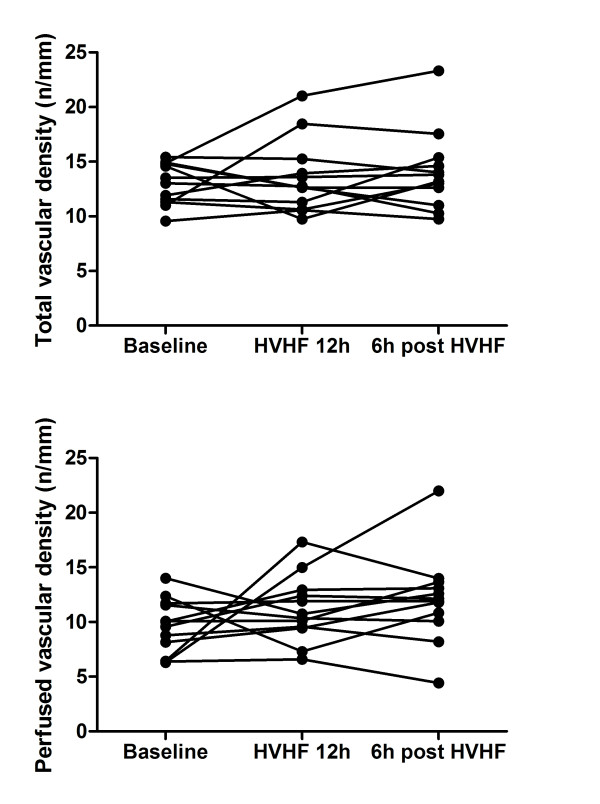
**Effects of high-volume hemofiltration (HVHF) on sublingual microvascular density**. The graphs present the individual evolution of total vascular density (upper graph) and perfused vascular density (lower graph) of small vessels (< 20 μm) at baseline, at the end of the 12-hour period of HVHF, and 6 hours after stopping HVHF. There was no significant change. Density is expressed as the number of vessels divided by the total length of the gridline in millimeters.

**Figure 2:**
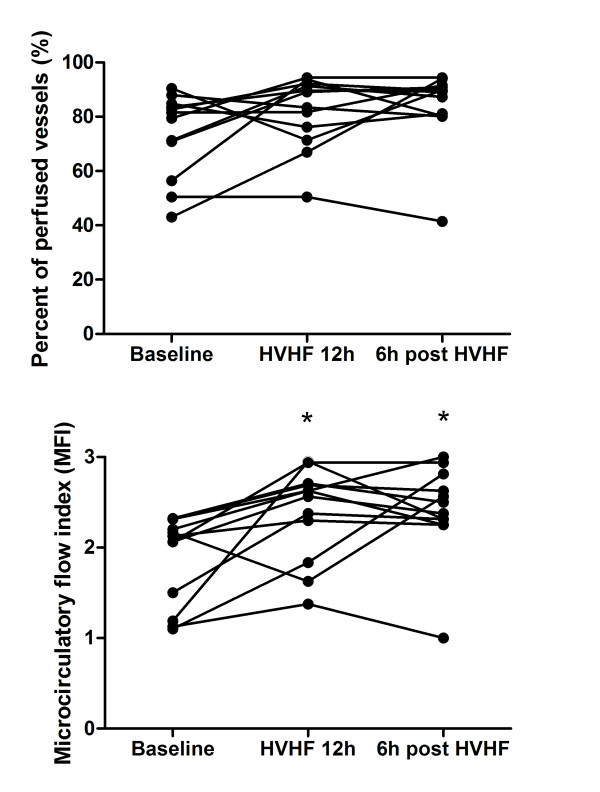
**Effects of high-volume hemofiltration (HVHF) on sublingual microvascular flow**. The graphs present the individual evolution of flow assessed by the percentage of perfused vessels (upper graph) and by the microvascular flow index (lower graph) of small vessels (< 20 μm) at baseline, at the end of the 12-hour period of HVHF, and 6 hours after stopping HVHF. *P < 0.05 compared with baseline.

Density scores (TVD and PVD) and Het MFI did not show any significant variation during the study (Figure 1 and Table 2). MFI significantly increased compared with baseline after 12 hours of HVHF and did not deteriorate after HVHF was stopped. In parallel, there was a trend to increased PPV during HVHF (Figure 2 and Table 2). Interestingly, three of the four patients with the worst MFI (less than 2) had a significant improvement after 12 hours of HVHF.

**Figure 3:**
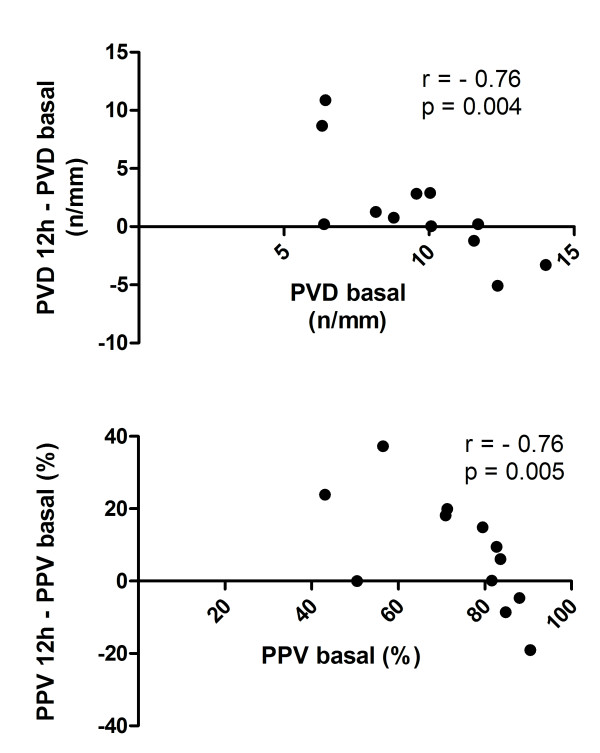
**Relationship between baseline sublingual microcirculatory parameters and their change during the 12-hour high-volume hemofiltration (HVHF)**. The upper graph shows a significant correlation between baseline values of perfused vascular density (PVD) and their variation during the 12-hour HVHF. The lower graph shows a similar correlation between the baseline values of the percentage of perfused vessels (PPV) and their variation during the 12-hour HVHF. Both PVD and PPV were calculated for small vessels (< 20 μm). Density is expressed as the number of vessels divided by the total length of the gridline in millimeters.

We looked for correlations between microcirculation at baseline and the relative changes occurring during the 12-hour HVHF. For PVD and PPV, there was a strong negative correlation such that patients with the worst scores at baseline had the largest improvements during the 12-hour HVHF (Figure 3). For TVD, MFI, and Het MFI, there was no significant correlation between baseline values and their relative changes during HVHF. In addition, we looked at correlations between microcirculatory changes and changes in hemodynamic and perfusion parameters (Table 3). There was no significant correlation.

**Table 3:** Correlations between variations in microcirculatory scores observed during high-volume hemofiltration and variations in systemic hemodynamic and organ dysfunction parameters

	MAP	NE	Lactate	CI	SmvO_2_	IDO_2_	IVO_2_	O_2_ER	SVRI	SOFA
TVD	−0.01	0.25	0.34	−0.14	0.31	0.12	0.10	−0.09	0.10	0.23
PVD	0.18	0.22	0.30	−0.08	0.22	0.13	0.12	−0.06	0.01	0.40
PPV	0.24	0.02	0.30	0.06	0.27	0.15	−0.15	−0.16	0.09	0.47
MFI	0.40	−0.03	0.25	0.24	−0.01	−0.02	0.17	−0.09	−0.13	0.37

Variations for each parameter were calculated as the difference between values at 12 hours of high-volume hemofiltration and values at baseline. Data correspond to correlations (*r *values) obtained either by Pearson coefficient (total vascular density [TVD], perfused vascular density [PVD], and proportion of perfused vessels [PPV]) or by Spearman's rho (microcirculatory flow index [MFI]). None of the correlations was statistically significant. CI, cardiac index; IDO_2_, oxygen delivery index; IVO_2_, oxygen consumption index; MAP, mean arterial pressure; NE, norepinephrine; O_2_ER, oxygen extraction ratio; SmvO_2_, mixed venous oxygen saturation; SOFA, Sequential Organ Failure Assessment; SVRI, systemic vascular resistance index.

## Discussion

In the present study, we found no deterioration of sublingual microcirculation during HVHF, despite an increase in systemic vascular resistance in patients with severe hyperdynamic septic shock. Furthermore, microcirculatory flow index significantly improved during HVHF, whereas PPV showed the same trend, which did not reach statistical significance. These effects seem to be more marked in patients with more impaired basal microcirculation.

Several experimental and clinical studies have suggested that HVHF can be an effective rescue therapy in refractory septic shock, stabilizing hemodynamics, decreasing vasopressor requirements, and improving lactate clearance [1,2,15]. This is the first study that explores the effects of HVHF on microcirculation in patients with septic shock. We observed an increase in sublingual microcirculatory blood flow during HVHF. Interestingly, this increase occurred despite an increase in SVRI and a trend to decreased cardiac output. One of the theories proposed to explain microcirculatory alterations in sepsis is the presence of shunt. The observation of increasing microcirculatory blood flow paralleled by increasing vascular resistance and decreasing cardiac output may be explained by a reversal of shunt.

The underlying mechanisms involved in the changes observed on hemodynamics and microcirculation are unclear. HVHF may remove some inflammatory mediators involved in the hemodynamic collapse of refractory septic shock from the blood compartment or the extravascular space [16]. Owing to its broad theoretical physiologic effects, HVHF could potentially influence several microcirculatory parameters and improve microcirculatory derangements in septic shock. However, because of the uncontrolled design of our study, we cannot rule out that changes observed on hemodynamics and microcirculation were not related to HVHF. The changes might correspond to the natural evolution of septic shock after initial resuscitation, as shown by Sakr and colleagues [17], or occur as the result of other cointerventions such as ongoing fluids or a strict hemodynamic management.

There has been controversy about the role of systemic hemodynamic variables on microcirculation [18,19]. Theoretically, arterial pressure could influence microcirculatory flow if autoregulation is altered, or norepinephrine could induce a decrease in microcirculatory flow secondary to vasoconstriction. Trzeciak and colleagues [19] found a positive correlation between MAP and sublingual microcirculatory blood flow in septic shock patients during the early phase of resuscitation. However, two elegant physiologic studies performed in septic shock patients have shown that arterial pressure changes induced by changing norepinephrine doses do not influence sublingual MFI across a large range of arterial pressures and norepinephrine doses [20,21]. In the present study, MAP increased from 67.5 ± 4.6 mm Hg at baseline to 74.5 ± 6.8 mm Hg at 12 hours of HVHF, but we found no significant correlation between changes in MAP and changes in MFI during the 12-hour HVHF. We also looked for correlations between changes in other systemic hemodynamic variables and changes in sublingual microcirculation during HVHF and found no significant correlation. Therefore, our data do not support the possibility that the increase in MFI observed was induced by changes in systemic hemodynamics.

Previously, an elegant experimental study compared the effects of standard hemofiltration versus HVHF in a porcine model of hyperdynamic sepsis [10]. Although HVHF was associated with an improvement in global hemodynamics, no beneficial effect on microcirculatory flow, hepatosplanchnic hemodynamics, cellular energetics, endothelial injury, or systemic inflammation could be observed. Unfortunately, the model induced only mild to moderate disturbances in hemodynamics and microcirculatory flow and therefore the condition did not represent severe septic shock.

Until now, only a few uncontrolled small studies have evaluated the hemodynamic effects of HVHF in patients with septic shock. Honore and colleagues [1] showed that HVHF responders improved cardiac output and systemic hemodynamics in a series of patients with hypodynamic septic shock. In our previous report involving only patients with hyperdynamic septic shock [2], we found that MAP increased mainly because of an increase in SVRI. However, an improvement in MAP at the expense of an increase in SVRI may not necessarily be beneficial in terms of microcirculatory flow [21], perfusion parameters [22], or survival [4]. The non-selective nitric oxide synthase inhibitor 546C88 induced a strong pressor effect in patients with septic shock, but unfortunately this effect was associated with higher incidences of pulmonary hypertension, systemic arterial hypertension, and heart failure; a decreased cardiac output; and a higher mortality [4]. Therefore, our results may be relevant since they suggest that the potential beneficial hemodynamic effect of HVHF is not at the expense of microcirculatory flow.

It is rather surprising that only 4 of 12 patients exhibiting severe septic shock presented the low MFI of less than 2. This observation is consistent with recent data from Dubin and colleagues [20] and Jhanji and colleagues [21], who found mean basal MFI values of 2.1 ± 0.7 and 2.3 ± 0.4, respectively. In fact, in the former study, only 4 of 22 patients with septic shock exhibited an MFI of less than 2. This is in sharp contrast with the data of Trzeciak and colleagues [19], who reported MFI values of less than 1.5 early after emergency room or ICU admission. It appears that MFI values, resembling what happens with ScvO_2_, are very low in pre-resuscitated patients but may improve after aggressive resuscitation, except in refractory patients who are dying.

We found a negative correlation between the severity of basal microcirculatory derangements and their change after a 12-hour HVHF session. Similar observations have been reported by other authors when studying the effect of different interventions on microcirculatory dysfunction in septic patients. Dubin and colleagues [20] assessed the effects of increasing MAP over microcirculatory dysfunction and found that changes in perfused capillary density correlate inversely with basal values. Sakr and colleagues [17] showed that changes in capillary perfusion after red blood cell transfusion correlate negatively with baseline capillary perfusion. At this moment, we have no clear explanation for these findings, but it appears that different interventions aimed at improving microcirculatory flow may be more effective in patients with more severe basal derangements.

The present study has several limitations. First, it includes a small number of patients. In our current septic shock management algorithm, HVHF is a rescue therapy. As reported elsewhere [12], the strict application of our protocol has led to an improvement in outcome, and therefore only 20% of septic shock patients are eligible for this intervention. Since only hyperdynamic septic shock patients with norepinephrine requirements of at least 0.3 μg/kg per minute and progressive hyperlactatemia were included in this study, we recruited only 1 patient every 45 days. This fact precluded the inclusion of a larger number of patients. Second, we did not include a control group. This limitation is shared by several studies addressing the impact of conventional therapies on microcirculation [6-8,23]. In our case, this was an observational pilot study and therefore a control group was not considered. However, we acknowledge the advantage of having a control group for future studies. In fact, the only randomized controlled trial involving microcirculatory dysfunction, which compared nitroglycerin versus placebo in patients with septic shock, found that MFI improved over time in both groups in the setting of a strict-background common-resuscitation protocol [9]. Third, our study protocol considered microcirculatory reassessment only after completing the standard 12-hour HVHF procedure, and thus we could have missed earlier effects. We selected a 12-hour design for two reasons: (a) the first couple of hours after starting HVHF are characteristically unstable, and patients are subjected to frequent fluid challenges or vasopressor titration that preclude a clear interpretation of microcirculatory changes; and (b) we were interested in evaluating the full effect of a 12-hour pulse HVHF session. Finally, it is still unclear whether the sublingual microcirculation is representative of other organs [24,25], so additional studies are necessary to assess the impact of HVHF over other microvascular beds.

## Conclusions

The use of HVHF as a rescue therapy in patients with severe hyperdynamic septic shock is not associated with deterioration of sublingual microcirculation, despite the increase in systemic vascular resistance. For the clinician, this suggests that the arterial pressure and SVRI increases that are usually observed during HVHF are not at the expense of microcirculation. Furthermore, patients with the lowest values of sublingual microcirculatory blood flow seem to improve in this respect during HVHF. However, randomized controlled studies with HVHF in septic shock are required to confirm and better define the physiologic effects of HVHF on hemodynamics and perfusion.

## Key messages

• During high-volume hemofiltration in patients with hyperdynamic septic shock, there is no deterioration of sublingual microcirculation, despite an increase in systemic vascular resistance.

• Sublingual microcirculatory blood flow may even increase during high-volume hemofiltration.

• Septic shock patients with the lowest values of sublingual microcirculatory blood flow at baseline exhibit a more pronounced improvement during high-volume hemofiltration.

## Abbreviations

CI: cardiac index; Het MFI: heterogeneity of microvascular flow index; HVHF: high-volume hemofiltration; ICU: intensive care unit; MAP: mean arterial pressure; MFI: microvascular flow index; NTSC: National Television System Committee; PP: pulse pressure; PPV: proportion of perfused vessels; PVD: perfused vascular density; ScvO_2_: central venous oxygen saturation; SDF: side dark field videomicroscopy; SmvO_2_: mixed venous oxygen saturation; SVRI: systemic vascular resistance index; TVD: total vascular density.

## Competing interests

The authors declare that they have no competing interests.

## Authors' contributions

CR, GH, and AB conceived of the study, participated in its design and coordination as well as data analysis, and drafted the manuscript. CG participated in image and data analysis. MA and PD conceived of the study, participated in data analysis, and helped to draft the manuscript. All authors read and approved the final manuscript.
